# Contra-lateral Auditory Brainstem Responses in Dyslexia

**Published:** 2016

**Authors:** Mehdi AKBARI, Mohammad Taghi JOGHATAEI, Akram NOORBAKHT, Mohammad Sadegh JENABI

**Affiliations:** 1 Cellular and Molecular Research Center (CMRC), Iran University of Medical Science (IUMS), Tehran, Iran; 2 Department of Audiology, Iran University of Medical Science (IUMS), Tehran, Iran; 3 Department of Speech Therapy, Iran University of Medical Science (IUMS), Tehran, Iran

**Keywords:** Auditory brainstem responses, Dyslexia, Contralateral, Ipsilateral

## Abstract

**Objective:**

Developmental care comprises a wide range of medical and nursing interventions used in the neonatal intensive care unit (NICU) to mitigate and reduce stressors affecting preterm or ill neonates. Because patient satisfaction survey is a valuable quality improvement tool, we aimed to develop and test the psychometric properties of a tool for measuring parent satisfaction of developmental care in the NICU.

**Materials &Methods:**

In this psychometric methodological study, the item pool and initial questionnaire were designed based on a comprehensive literature review and exploring NICU parent satisfaction questionnaires. The validity of the designed questionnaire was determined using face, content (qualitative and quantitative), and construct validity. Exploratory factor analysis was performed using responses from 400 parents of infants hospitalized in the NICUs of 34 hospitals in 2015 in Tehran, Iran. The reliability of the questionnaire was identified using Cronbach’s alpha and stability measures.

**Results:**

The initial questionnaire was designed with 72 items in five domains. After testing the face validity, 3 items were omitted. The results of validity testing were acceptable. The exploratory factor analysis was performed on 69 items, and 5 factors (care and treatment with 20 items, information with 15 items, hospital facilities with 9 items, parental education with 7 items, and parental participation with 8 items) were extracted. The reliability was supported by high internal consistency (α = 0.92).

**Conclusion:**

This questionnaire could be valid and reliable tool for measuring parents’ satisfaction.

## Introduction

Learning disability is a term that being used for complex learning problem that caused by mental disorder. There is a difference between learning disability and learning difficulty. People with learning difficulty can use routine techniques, while a person with learning disability needs special education and interventions, depending on the type of disability. The most common type of learning disability is dyslexia which is known recently as specific learning disability (SLD) as well. Other forms of SLD are dyscalculia, dysgraphia, and short term memory dysfunction. SLD has neurological bases. Dyslexia may be defined as a specific learning disability that is neurological in origin and characterized by a difficulty with accurate reading fluency and poor decoding and spelling, resulting from a deficit in the phonological component of language(1).

Dyslexia is a prevalent problem characterized by difficulties in reading, writing and spelling despite having normal or above moderate intelligence. Until now, despite many researches, the exact origin of dyslexia is unknown.

Dyslexia is a neurological disorder and is thought to affect 5% to 10% of schoolchildren (2). Other researchers have mentioned that dyslexia is a neurological disorder affecting literacy skills in approximately 5%–10% of school-aged children(3, 4). The brainstem structures are very important for perception and signal processing in noisy environments in auditory system. Processing in subcortical structures involves an interaction between sensory and cognitive systems. Besides, these structures have a special role by feed forward and feedback of this pathway (5). The connection between cortex and subcortical system form the basis for such interaction related top-down control (6). In human study, the neural response of auditory system to complex stimuli can be measured from lower levels of the nervous system such as brainstem. The auditory brainstem responses (ABR) technique is a sensitive method to evaluate this pathway and actually for detection of complex harmonic characteristics of speech (7). The human auditory brainstem is dynamic in nature, according to this fact some researcher (8) who have examined neural plasticity at the level of brain stem, have used proficient group of musicians and controls. Auditory brainstem system is sensitive to auditory experience.

Reading is a complex skill, and in order to adequate these skills, coordination of auditory system, visual, phonetic and lexical codes is required (9). Neuro-imaging and electrophysiologic studies have demonstrated that reading involves an extensive network of regions in the cerebral cortex(10, 11). Neurobiological study of dyslexia have attempted to find the role of these different cortical portions, but there are many aspects of this issue that remain unknown about function bases of reading skill and reading disorders.

For better evaluation and intervention of this area we need a team of specialists such as audiologist, speech therapist, neurophysiologist, neuro-pathologist, and educational scientist.

During the past years many researchers have concentrated on different scientific theoretical and practical areas of assessment of dyslexic children. The idea that dyslexia may have a brain abnormality is not so new. Scottish ophthalmologist James Hinshelwood and Morgan both emphasized on certain neurological syndrome of “visual word blindness” (12, 13). Damage of the left inferior parieto-occipital region impairs reading and writing skills (14). Reading and writing defects could be due to same parietal region which has been damaged (12). These hypotheses are confirmed during brain description of a dyslexic boy who died because of brain hemorrhage due to a vascular malformation(15). Pathological findings showed a series of brain malformations in the cortical gyri of the left inferior parietal region. Another line of neurological defect has followed by Orton as the ‘founding father’ of the lateralization theory of dyslexia (16). This idea has proposed by others by which the lateralization of language to the left hemisphere would be delayed in dyslexic cases (17). It seems that this area of the brain which is related to learning and reading might not be completely developed. Several investigations have confirmed this theory as the brain asymmetry using some dichotic tests (17).

According to world health organization (WHO) definition, dyslexic children’s have specific and significant impairment in reading and writing, in general intelligence and sensory acuity. Moreover, several associated problems such as oral language acquisition (dysphasia), writing problem (dysgraphia), mathematical problem (dyscalculia), motor coordination (dyspraxia), temporal orientation (dysphonia), and attentional abilities (Hyperactivity and attention deficit) have been reported among dyslexic children (18). 

Brain imaging studies have also demonstrated that reading abilities begins initially as a phonological process. In these phases of learning, it is the neural structures for speaking that are especially active. As reading skill improves, an area in visual cortex named “visual word form area” (VWFA) becomes active (19).

It is very close to the visual cortex that is active during picture naming. This area also is active during nonsense word reading, the VWFA is store orthographyphonology. Dyslexia shows selective under activation of phonological area of brain, but targeted phonology based intervention makes better levels of activation in VWFA (20).

Dyslexic children have processing disorder, which are unable to correlates between brainstem and cortical auditory processes (21). Cortical abnormality in most of the cases with learning problem is possibly a result of inputs to the thalamo-cortical circuitry which may be a result of decreased processing and or transfer of data at the lateral lemniscus and inferior coliculus (22). Processing in the lateral lemniscus and/or inferior colliculus is clearly consist in all learning problems. signals disturbed at the lower level of brainstem can affects in auditory cortex (22). Auditory brainstem responses are special assessment tools to quantify and qualify auditory system. In terms of determining of hearing degree, type of hearing loss, configuration of hearing loss, screening of neonate and other special hard to test populations like mental retards, attention deficits and sensory and motor delay. It is now possible to assess the human brain electrical activities that are generated through the auditory system from cochlea to cortex. Auditory brainstem response contains a series of nuclei that receives input from the acoustic nerve and processes this signal as it enters the neocortex. An important structure in the midbrain is inferior colliculus (IC), because it is a station between ascending projections from the lower brainstem nuclei and ascending projections to the thalamus. It is because of its unique arrangement of converging ascending and corticofugal projections (23). The ABR is a noninvasive measurement of synchronous electrical activities. In response to an acoustic signal, a series of waves measures at the scalp. This measurement provides information about the function of brainstem nuclei along the ascending auditory pathway (24). Monaural mode of assessment of ABR has remained as a useful tool for evaluation bilateral or unilateral disorders (25- 27). Dyslexia has neurological bases, and on the other hand, auditory brainstem response depends on a high degree of synchronized firing between neurons that can reflect neural synchrony to speech, elements of sounds, and interaction between brainstem and auditory cortex (28).

Our hypothesis was the fact that contralateral ABR provides more information on the dyslexic brainstem and plays a role in signal possessing. Hence, in order to achieve these goals, we used IPSI and contra lateral ABR on cases with dyslexia. Brainstem is entrance gate of auditory and speech information, and auditory brainstem responses is a useful tool for assessment and evaluation of this pathway. It is well known that each wave has special region. Wave I originates from the ipsilateral auditory system at the entry to the brainstem. Wave III is near the ipsilteral cochlear nucleus and also is associated with superior olivary complex (28). Wave V is also associated with activity in the contralateral lateral lemniscus. This nucleus terminates in the inferior colliculus(29,30). Waves III and V have contralateral region. Both waves III and V have greater number of contra than ipsilateral.

## Materials & Methods

**Subjects**

Fifty two dyslexic cases (30 males and 22 females) aged between 8-10 years and 53 matched-controls were recruited (Table 1). All the cases had normal IQ or above moderate. Inclusion criteria were normal hearing thresholds, normal tympanometry and reflex, and also normal vision or near normal with glasses.

**Stimuli and recording**

Brainstem responses to click stimulus were collected according to widely used procedures as described in detail by Hood and Jacobson (24). An EB NEURO device was used to collect all physiological data. The brainstem responses to clicks were elicited by alternating polarities. The test stimuli were presented to each ear through TDH 39 earphones at an intensity of 75 dB nHL. Recordings were made with silver silver chloride electrodes, impedance less than 3k. Responses were separately recorded from Cz, Fz and Pz to ipsilateral and contralateral A1and A2 earlobe. Responses were collected at a rate of 16.1/s. For the click, a 10 ms recording time window was used (including a 0.8 ms pre-stimulus period), and responses were on-line filtered from 150 to 2000 Hz. Click stimuli were applied to the right ear and at the same time ipsilateral and contralateral responses were recorded. The same method was done for left ear as well. Responses recorded in this study were thought to arise primarily from the auditory brainstem because of the filter characteristics and stimulation rates that were used.

This study was confirmed by Ethics Committee of the Iran University of Medical Sciences and written consent form was taken from each patient and their parents.

**Data analysis**

Auditory brainstem responses measure is as reflecting the synchronization of the summation of neural activity. Specifically, the wave V is thought to reflect lateral lemniscus input to the inferior colliculus (wave V), and subsequent dendritic processing in the inferior colliculus (wave Vn). For the analysis of click stimulus, absolute latency and peak amplitude of wave V for each subject were detected. As it is typically used in clinical evaluations, peak V was detected as the final data point on the wave form before the negative slope that follows the wave.

The contralateral ABR recording in a two channel setup can be useful for neuro-diagnostic purposes. In many cases, the contralaeral recording shows a clear split between waves IV and V, but it should have the same latencies or in normal limit as the ipsilateral recording. Data were analyzed using SSPS version 21 (Chicago, IL, USA).

## Results

Data was collected in both control and tested group, according to previous normalized data. We used the inter-aural latency difference less than 0.2 milli seconds. 

All controls were in normal limit. Normative data were collected and the mean values of absolute latencies for ipsilatral left ear in the rate of 16.1 were 1.77 ms, 3.69 ms and 5.65 ms respectively. For the right ear, these values were 1.76 ms, 3.71 ms and 5.64 ms respectively. 

Mean values of inter-peak latencies of wave I-III, III-V, I-V were 1.92 ms, 1.96ms and 3.88ms respectively for left ear. For the right ear, values were 1.95 ms, 1.93 ms and 3.88 ms respectively. In comparison between contra and ipsilatral recording, increasing mean value latency in left and right ear was 0.18 ms. 

In dyslexic group, mean values of ipsilateral in the rate of 16.1 for left ear was 5.70 ms.For the right ear, it was 5.69 ms. Contralateral for the left ear was 5.73 ms and for the right ear was 5.72 ms. Mean value of ipsi and contralateral showed no significant association.

While 19 dyslexic cases in contralateral recording had abnormal latency more than 2 ms in comparison with control group.

## Discussion

Temporal information encodes by neural activity in the brain. According to physiologic and anatomic findings, it has been suggested that this information is processed differently in the brain, right hemisphere for syllabic and the left hemisphere for processing phonemic-rate acoustic information (7).

In a study on the functional role of the inferior colliculus, suggested that, concentrated only on the hemisphere, ignoring the basic data in auditory system such as the Inferior colliculus and lateral lemniscus (32), and superior olivary complex is also involved in computing sound localization (33). ABR routinely uses ipsilaterally. Contralateral is useful for distinguishing between waves IV and V. Contralateral ABR can be used for measurement of developmental changes in the infant auditory system (34). 

In the present study, two integrated methods namely IPIS and contralateral were used for assessment of brainstem functions. Twenty seven percent of dyslexic children in contralateral recording had abnormal latency. Brainstem has important role in processing of language information. Applying ABR with special protocol could be useful for dyslexic cases. Comparison between low and high rate ABR have been used as neuropathy. We found that ABR is a unique technique for assessment of dyslexia. In another study showed that interaction between cortex and brainstem is very important in learning skills (35). The results of present study are in agreement with these hypotheses. But the important thing at this area is detection of suitable for this issue.

**In conclusion, **In this study to evaluate the function of the auditory brain stem response in dyslexic individuals, from two conventional methods ipsilatral and other methods contralatral were also used. In Ipsilatral auditory brainstem responses did not find significant difference in the test group with the control group. But in other method, results were significant compared to the control group.
